# Amino acid-mediated impacts of elevated carbon dioxide and simulated root herbivory on aphids are neutralized by increased air temperatures

**DOI:** 10.1093/jxb/eru439

**Published:** 2014-11-16

**Authors:** James M. W. Ryalls, Ben D. Moore, Markus Riegler, Andrew N. Gherlenda, Scott N. Johnson

**Affiliations:** Hawkesbury Institute for the Environment, University of Western Sydney, Locked Bag 1797, Penrith, NSW 2751, Australia

**Keywords:** Aboveground–belowground interactions, aphid, climate change, legume, root herbivore, simulated herbivory.

## Abstract

Changes in host plant quality, including foliar amino acid concentrations, resulting from global climate change and attack from multiple herbivores, have the potential to modify the pest status of insect herbivores. This study investigated how mechanically simulated root herbivory of lucerne (*Medicago sativa*) before and after aphid infestation affected the pea aphid (*Acyrthosiphon pisum*) under elevated temperature (eT) and carbon dioxide concentrations (eCO_2_). eT increased plant height and biomass, and eCO_2_ decreased root C:N. Foliar amino acid concentrations and aphid numbers increased in response to eCO_2_, but only at ambient temperatures, demonstrating the ability of eT to negate the effects of eCO_2_. Root damage reduced aboveground biomass, height, and root %N, and increased root %C and C:N, most probably via decreased biological nitrogen fixation. Total foliar amino acid concentrations and aphid colonization success were higher in plants with roots cut early (before aphid arrival) than those with roots cut late (after aphid arrival); however, this effect was counteracted by eT. These results demonstrate the importance of amino acid concentrations for aphids and identify individual amino acids as being potential factors underpinning aphid responses to eT, eCO_2_, and root damage in lucerne. Incorporating trophic complexity and multiple climatic factors into plant–herbivore studies enables greater insight into how plants and insects will interact in the future, with implications for sustainable pest control and future crop security.

## Highlight

Multiple abiotic factors can combine to alter crop quality and rates of herbivore attack. Aphids benefit from elevated CO_2_ and root damage, but these effects are neutralized by increased temperatures.

## Introduction

Host plant quality and suitability for herbivorous insects shapes the extent to which plants are attacked (Bernays and Chapman, 1994), and sudden improvements in host plant quality can lead to pest outbreaks (Barbosa *et al.*, 2012). Biotic and abiotic factors often induce changes in secondary metabolites that might deleteriously affect herbivores (Iason *et al.*, 2012), but these can also change the nutritional quality of plants, leading to increased susceptibility of plants to herbivores (White, 1984; Johnson *et al.*, 2009). This is particularly true for mobile herbivores that have short generation times and respond quickly to changes in host plant nutrition, such as aphids (Dixon, 1998; Douglas, 2003). In particular, aphids respond to changes in the amino acid quality of phloem sap, which has been shown to be affected by both global climate change (Harrington *et al.*, 1995; Newman, 2003; Pritchard *et al.*, 2007; Sun and Ge, 2011) and root herbivory (Johnson *et al*., 2012 2013). To the authors’ knowledge, how these factors operate and interact remains largely unknown. In this study, experiments were carried out to examine how elevated temperature (eT) and atmospheric carbon dioxide concentrations (eCO_2_), as well as simulated root herbivory, acted alone and together to affect foliar amino acids and plant susceptibility to aphids.

Future CO_2_ concentrations (from current levels of 400 μmol mol^–1^ to >550 μmol mol^–1^ by 2050) are likely to be accompanied by increased temperatures (1–4 °C within this century) and, while a number of studies have observed effects of eCO_2_ on aphid populations, the combination of eT and eCO_2_ has received little attention (Newman, 2003; Himanen *et al.*, 2008; Murray *et al.*, 2013). In general, eCO_2_ is expected to increase plant growth by accelerating rates of photosynthesis, which reduces tissue quality and increases the carbon to nitrogen (C:N) ratio (Robinson *et al.*, 2012). This can be exacerbated when increased plant growth leads to N limitation (Rogers *et al.*, 2009). Legumes, such as lucerne (*Medicago sativa* L.), can, however, avoid N limitation and maintain tissue N concentrations under eCO_2_ by enhancing biological N fixation (Soussana and Hartwig, 1995; Johnson and McNicol, 2010). This can alter the dynamics of foliar-feeding aphids (Douglas, 1993), which tend to perform better on plants with higher N (Nowak and Komor, 2010; Sun and Ge, 2011) and amino acid concentrations (Ponder *et al.*, 2000; Karley *et al.*, 2002; Guo *et al.*, 2014). In contrast, eT may combat eCO_2_ effects on plant nutrient quality by decreasing biological N fixation, often associated with a low tolerance of N-fixing rhizobial bacteria to increased temperatures (Zahran, 1999; Whittington *et al.*, 2013), which ultimately decreases amino acid concentrations and aphid abundance (Ryalls *et al.*, 2013*a*).

Plants, in addition to being attacked by aboveground herbivores, often have to contend with root damage by herbivores, which may increase amino acid concentrations in the foliage and, in turn, make the plant more susceptible to aphid attack aboveground (Masters *et al.*, 1993; Johnson *et al.*, 2009). This might be different for legumes, however, since the roots are the source of N acquisition through biological N fixation. The general prediction that root herbivory usually has beneficial effects on aboveground aphids may also be altered by changes in herbivore synchronization (Erb *et al.*, 2011). In a meta-analysis, Johnson *et al.* (2012) identified the sequence of herbivore arrival (e.g. whether aphids arrive before or after roots are damaged) as the most influential factor affecting aboveground–belowground linkages. Root damage before aphids arrive may impair water uptake and lead to a stress-related accumulation of amino acids in the phloem, but crucially gives the plant a chance to recover and regain hydraulic properties (e.g. phloem turgor) which might allow aphids to capitalize on this. Root recovery is often rapid and can be overcompensatory (i.e. more root nodules are produced) in legumes (Quinn and Hall, 1992; Ryalls *et al.*, 2013*b*). Conversely, if aphids are already feeding on a plant when root damage occurs, root recovery and stress-related increases in foliar nutrients may be less likely because the plant remains in stress. This mirrors the pulsed stress hypothesis (Huberty and Denno, 2004), which posits that phloem-feeding insects exhibit poor performance on continuously stressed plants, yet respond positively when plant recovery is possible.

Using the model legume, lucerne, this study combines aboveground sap feeding by the pea aphid, *Acyrthosiphon pisum* (Harris), and simulated root herbivory in the form of root cutting, to characterize the effects of herbivore arrival sequence on aboveground–belowground interactions. While artificial herbivory may not mimic natural damage exactly (Blossey and Hunt-Joshi, 2003), it enables control of the type, timing, and intensity of damage, especially in complex systems such as this (Hjältén, 2004; Rogers and Siemann, 2004; Borowicz, 2010). Lucerne itself is the most important and widely grown forage legume worldwide (Small, 2011). The late 1970s saw the invasion of Australia by three aphid pests, including the pea aphid, which devastated lucerne stands in eastern Australia (Bouton, 2012). The development of resistant cultivars has helped to abate the aphid problem, but outbreaks driven by environmental factors and changes in the nature and intensity of trophic interactions (e.g. release from natural enemies) still occur (Zarrabi *et al.*, 1995; Reddy and Hodges, 2000; Humphries *et al.*, 2012; Ryalls *et al.*, 2013*a*).

**Fig. 1.**
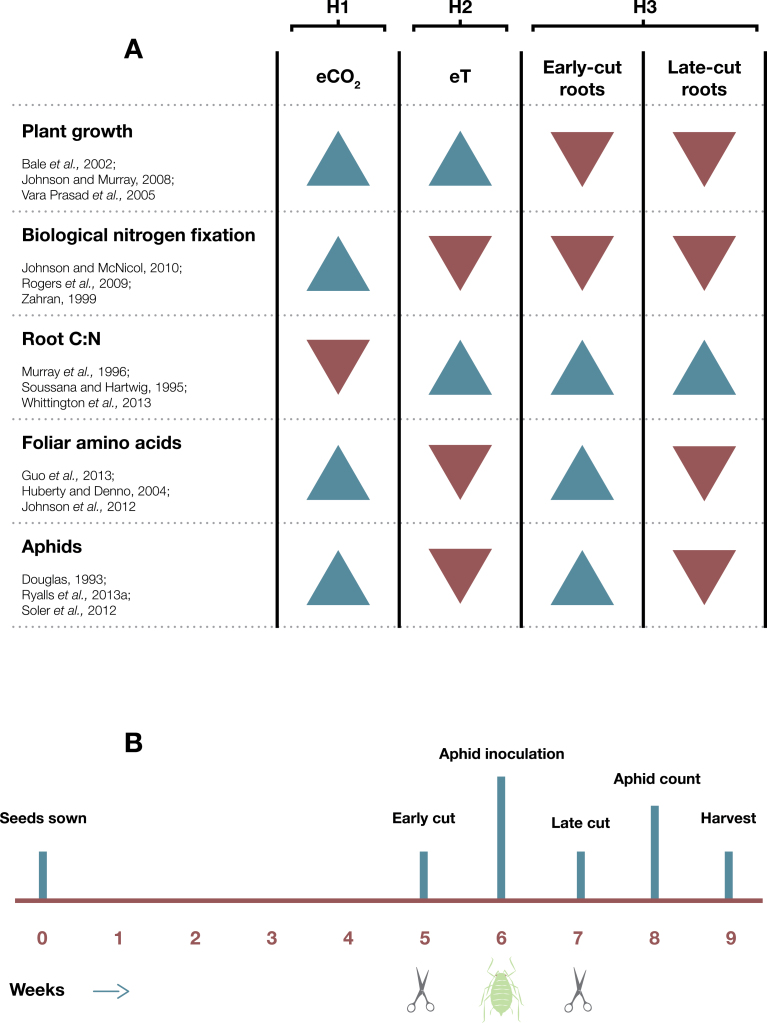
Hypothesized treatment effects of root cutting (before and after aphid infestation), elevated temperature, and CO_2_ on aphids and plant characteristics, with example references given as a basis for the hypotheses. H1, H2, and H3 refer to hypotheses (i), (ii), and (iii), respectively (A). Methodological timeline of experimental events (B).

This study specifically aimed to determine the effects of: eT and eCO_2_, individually and in combination, on lucerne growth (height and biomass), chemistry (root C:N and foliar amino acid concentrations and composition), and pea aphid abundance; and simulated root herbivory (root cutting before and after aphid arrival) on lucerne growth, chemistry, and pea aphids. It was hypothesized that: (i) eCO_2_ would increase lucerne growth and promote biological N fixation (i.e. decrease root C:N), which would increase foliar amino acid concentrations and ultimately aphid abundance. In contrast, (ii) eT would increase lucerne growth but reduce biological N fixation (i.e. increase root C:N), which would reduce foliar amino acid concentrations and aphid abundance; and (iii) root cutting would reduce lucerne growth and biological N fixation (i.e. increase root C:N) generally. Early root cutting would benefit aphids via increased foliar amino acid concentrations because stress and recovery of lucerne would be possible. In contrast, root cutting during aphid feeding would present above- and belowground stress, making recovery less likely, and would negatively impact aphids via decreased foliar amino acid concentrations. Hypothesized treatment effects of early and late root cutting, elevated temperature, and CO_2_ on aphids and plant characteristics are summarized in Fig. 1A.

## Materials and methods

### Glasshouse conditions

Experiments were conducted in four glasshouse chambers at the University of Western Sydney Hawkesbury campus, NSW (latitude –33.611141, longitude 150.745315) in which CO_2_ was maintained at either ambient CO_2_ concentrations, aCO_2_ (400 μmol mol^–1^), or at eCO_2_ (640 μmol mol^–1^), and temperature was maintained at either ambient temperature, aT (26/18 °C day/night on a 15:9 light:dark cycle), or eT (30/22 °C day/night on a 15:9 light:dark cycle). aT (26 °C) represents the average daily maximum temperature for Richmond, NSW over the last 30 years, and eT was consistent with the predicted maximum temperature increase of 4 °C for this region within this century (CSIRO, 2007–2014). Humidity was controlled at 55%, and no significant differences in light conditions were observed between chambers (Ryalls *et al.*, 2013*b*). Temperature, CO_2_, and humidity within glasshouse chambers were monitored continuously throughout the experiment, and plants were randomly reallocated among positions within chambers twice weekly to minimize potential within-chamber effects. The experiment itself was repeated three times, which was incorporated into statistical models to reduce the influence of pseudo-replication of CO_2_ and temperature treatments (Newman *et al.*, 2011).

### Aphid cultures

*Acyrthosiphon pisum* adults used in the experiment were taken from four established cultures, all originating from an individual parthenogenetic adult female collected from a local lucerne field in Richmond, NSW (latitude –33.609189, longitude 150.746953) in January 2013. Cultures were maintained on propagated lucerne plants in each of the four experimental chambers for at least six generations prior to the inoculation period.

### Experimental procedure

Lucerne plants (54 plants of cultivar ‘Sequel’) in each of the four chambers (aCO_2_, aT; eCO_2_, aT; aCO_2_, eT; and eCO_2_, eT) were distributed randomly among six treatments: aphids only (A), early cut no aphids (EC), early cut and aphids (ECA), late cut no aphids (LC), late cut and aphids (LCA), and control plants with no cutting or aphids (C), giving nine replicates of each treatment in each chamber. Five weeks after sowing, treatments EC and ECA were subjected to early root damage by severing the entire root system 35mm below the soil surface using a sharp steel blade inserted in a narrow opening cut into the plastic pot. One week later, treatments A, ECA, and LCA were inoculated with two teneral adult pea aphids. Organza bags (125×170mm) were secured around each plant to confine individual aphids to the plant. One week after aphid inoculation, treatments LC and LCA were subjected to simulated late root herbivore damage using the same technique as for those cut earlier. Aphids were counted and removed a week later (8 weeks after sowing). One week after aphids were removed, roots were separated from soil and shoots and all plant material was snap-frozen in liquid N and stored at –20 °C. Material was then freeze-dried and weighed to determine dry mass before chemical analysis. Plant heights (from ground level to the base of the highest leaf) were measured 5 (early cutting period), 6 (aphid inoculation period), and 9 (harvest period) weeks after planting (Fig. 1B).

### Chemical analyses

Four samples, selected at random, from each treatment combination per run were chosen for chemical analysis (288 samples overall). Freeze-dried roots and shoots were ball-milled for 90 s to a fine powder. Soluble amino acids were extracted from milled shoot samples (10mg) by shaking in 1.5ml of 80% ethanol for 20min at 50 °C. After centrifugation at 12 000 relative centrifugal force (rcf) for 5min to remove solids, 1ml of supernatant was removed and evaporated to dryness at 30 °C in a vacuum concentrator (Eppendorf). Amino acids were analysed by reverse-phase high-performance liquid chromatography (HPLC) in an Agilent 1260 Infinity HPLC system after pre-column derivatization using phenylisothiocyanate (PITC) (Crafts-Brandner, 2002; Reason, 2003). Derivatization steps were as follows: dried extracts were redissolved in 100 μl of coupling solution (acetonitrile:pyridine:triethylamine:water 10:5:2:3), vortexed, and redried under vacuum at 30 °C. They were then redissolved in 100 μl of coupling solution, followed by 5 μl of PITC, vortexed, and allowed to react for 5min at room temperature before redrying at 30 °C. A 100 μl aliquot of Milli-Q water was then added, vortexed, and redried at 45 °C. Samples were redissolved in 250 μl of analysis solvent (water:acetonitrile 7:2) and filtered through a 0.45 μm 4mm PVDF filter prior to HPLC analysis. HPLC was performed using an Agilent Poroshell 120 EC-C18 column (4.6×75mm, 2.7 μm) at a column temperature of 40 °C. The gradient, composed of eluent A (1 litre of Milli-Q water, 19g of sodium acetate trihydrate, 0.5ml of triethylamine, pH 5.7, 63.8ml of acetonitrile, 1.07ml of EDTA dipotassium salt 1g l^–1^ solution) and eluent B (acetonitrile:water 6:4), was as follows: 0–3.75min, 0–40% B; 3.75–4.5min, 40–80% B; 4.5–5.75min, 80–100% B; 5.75–7min, 100% B; 7–9min, 100–0% B. The flow rate was 1.4ml min^–1^. Amino acid derivatives were detected by ultraviolet absorbance at 254nm. Amino acid standards (5, 10, 15, 20 and 25 nmol) containing 17 amino acids (see below) and an internal standard (12.5 nmol l^–1^ norleucine) were used to calibrate the analysis. Nine essential amino acids (i.e. those that cannot be synthesized by insects *de novo*), namely arginine, histidine, isoleucine, leucine, lysine, methionine, phenylalanine, threonine, and valine (Morris, 1991), and eight non-essential amino acids (alanine, aspartic acid, cysteine, glutamic acid, glycine, proline, serine, and tyrosine) were detected using this method. Foliar amino acid composition was measured as it has been demonstrated to be a reliable indicator of phloem amino acid composition (Winter *et al.*, 1992; Johnson *et al.*, 2014). Root C and N concentrations of 4–6mg of milled root samples were determined using a Carlo Erba CE1110 elemental analyser with thermal conductivity and mass spectrometric detection (of N_2_ and CO_2_). The percentage of N and C in the sample was calculated by comparison with known standards.

### Statistical analyses

Linear mixed effect models were produced using the nlme (v3.1–109) and lme4 (v0.999999-2) statistical packages for aphid abundance and aphid colonization, respectively, in the R statistical interface v3.0.1. Models described the effects of simulated root herbivory, eT, and eCO_2_ on aphids. The fixed terms included were plant-damage treatment (C, A, EC, ECA, LC, and LCA), temperature (26 °C and 30 °C), and CO_2_ (ambient and elevated), as well as the interactions between these terms. The random terms included were experimental run, with chamber as a nested factor, to account for pseudoreplication and chamber effects that could confound temperature and CO_2_ effects. The dependent variable ‘aphid abundance’ was log+1 transformed to normalize the model-standardized residuals. Aphid colonization (i.e. the proportion of plants hosting aphids at harvest) was analysed using a binomial error structure. Models were reduced by deleting non-significant fixed terms in a stepwise manner using Akaike information criterion (AIC) values and associated *P*-values taken from analysis of variance (ANOVA) model comparisons. Post-hoc Tukey’s tests and the R package LMERConvenienceFunctions were used for pairwise comparisons of means for treatment and interaction effects. The effects of simulated root herbivory, aphid presence, eT, and eCO_2_ on lucerne shoot biomass, height, and root biomass were also analysed with mixed models using nlme in R. Dependent variables were log-transformed to normalize model residuals. The random terms included were run/chamber and fixed terms were reduced in a stepwise manner, as above. Temperature, CO_2_, and plant-damage treatment effects on total, essential, and individual amino acid concentrations were also analysed using linear mixed models with log-transformed dependent variables to normalize residuals. Similar models with log-transformed dependent variables were used to determine the effects of temperature, CO_2_, and plant-damage treatment on root %N, C, and C:N. Principal components analysis (PCA) and permutational multivariate analysis of variance (PERMANOVA) were used to explore the impacts of eT, eCO_2_, and plant-damage treatment on amino acid composition. Groupings of individual amino acids were determined using a correlation matrix, and PERMANOVA results were compared with those obtained from individual mixed models.

## Results

### Impacts of eT and eCO_2_

**Fig. 2.**
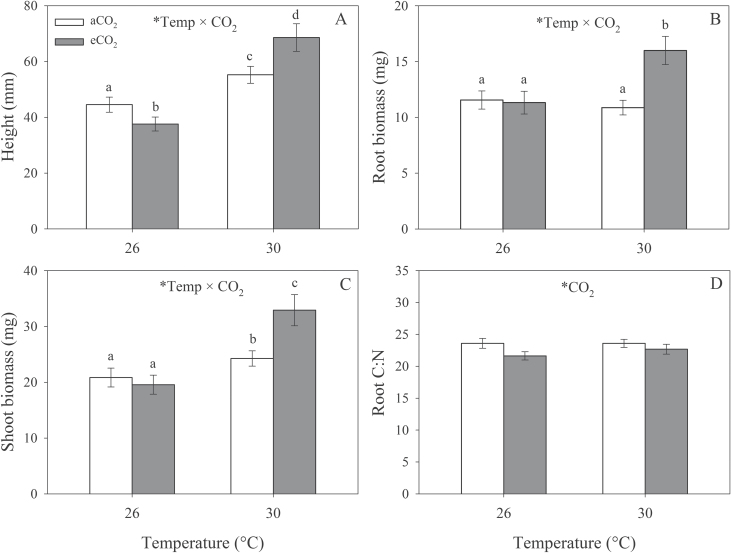
Effects of elevated temperature and CO_2_ on plant height (A), root biomass (B), shoot biomass (C), and root C:N (D). Mean values (± SE) are shown. Statistically significant effects are indicated by * (*P*<0.05). Bars with the same letters were not significantly different (*P*<0.05).

Temperature in interaction with CO_2_ had a significant effect on lucerne height (*F*_1,6_=7.80; *P*=0.032), root biomass (*F*_1,6_=21.76; *P*=0.003), and shoot biomass (*F*_1,6_=6.45; *P*=0.044). At 30 °C, plants were taller under eCO_2_, whereas, at 26 °C, plants were shorter under eCO_2_ (Fig. 2A). Lucerne root and shoot mass increased under eCO_2_ at 30 °C, but no effects of eCO_2_ were observed at 26 °C (Fig. 2B, C). eCO_2_ decreased root C:N significantly (*F*_1,8_=7.90; *P*=0.023) (Fig. 2D) but did not significantly increase root %N (*F*_1,8_=4.55; *P*=0.065) or decrease root %C (*F*_1,8_=2.59; *P*=0.146) individually.

**Fig. 3.**
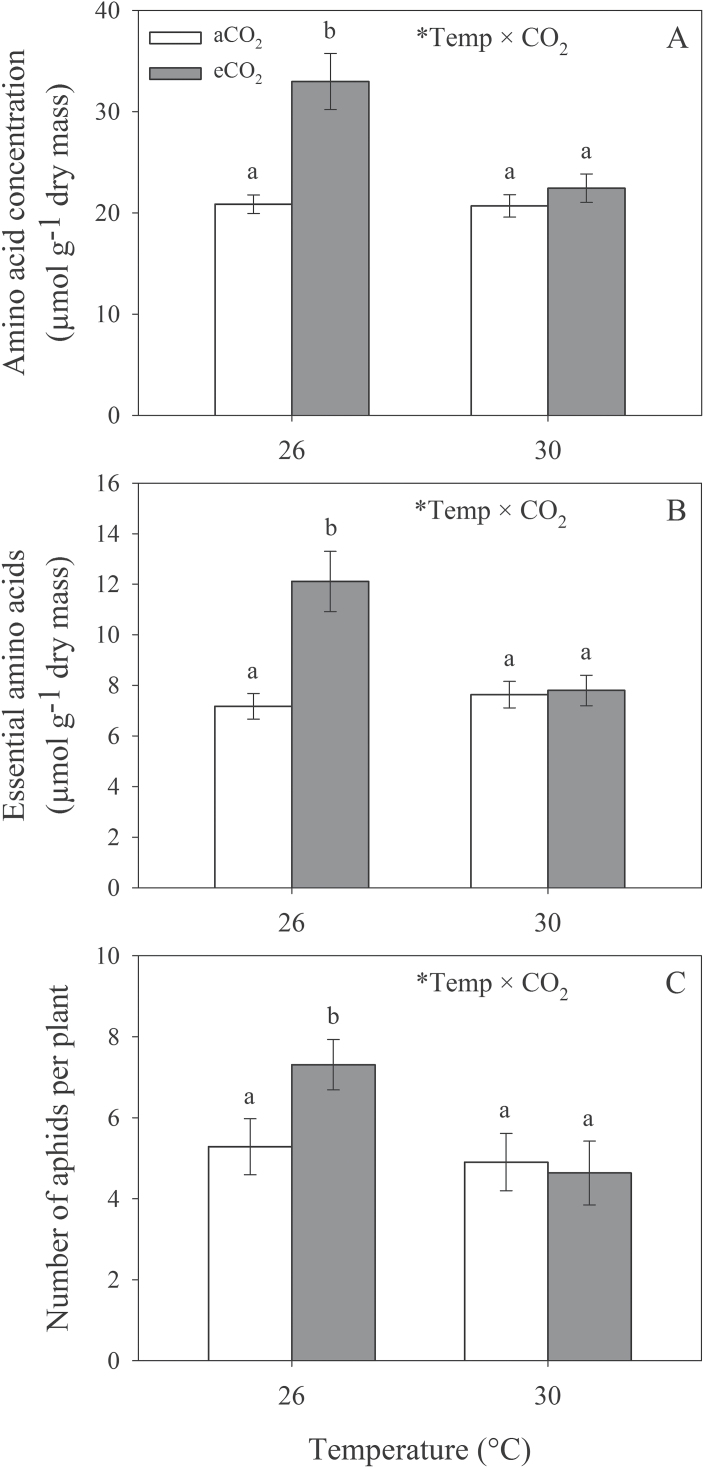
Effects of elevated temperature and CO_2_ on total amino acids (A), essential amino acids (B), and aphid abundance (C). Mean values (± SE) are shown. Statistically significant effects are indicated by * (*P*<0.05). Bars with the same letters were not significantly different (*P*<0.05).

Significant interactions of temperature and CO_2_ were also observed for total (*F*_1,287_=7.63; *P*=0.006) and essential amino acid concentrations (*F*_1,292_=10.072; *P*=0.002), as well as aphid abundance (*F*_1,6_=6.45; *P*=0.044), whereby eCO_2_ significantly increased total amino acids (Fig. 3A), essential amino acids (Fig. 3B), and pea aphid numbers (Fig. 3C) at 26 °C but not at 30 °C. Results from individual amino acid analyses (Table 1; Supplementary Table S1 available at *JXB* online) showed that eT decreased concentrations of arginine, aspartic acid, glutamic acid, and histidine, and eCO_2_ increased concentrations of alanine, glutamic acid, leucine, lysine, phenylalanine, proline, and serine. The interaction of eT and eCO_2_ affected glycine and glutamic acid, whereby concentrations of both amino acids increased under eCO_2_, but only at 26 °C.

**Table 1.** Summary of final model statistical analyses of individual amino acid responses to plant damage (Treatment), temperature, and CO_2_ treatments

Amino acid	CO_2_		Temperature		Treatment		Temperature×CO_2_		Treatment× Temperature	
	*F*_1,6_	*P*	*F*_1,6_	*P*	*F*_5,264_	*P*	*F*_1,6_	*P*	*F*_5,264_	*P*
Alanine (Ala)^1^	28.26	0.002	–	–	3.48	0.005	–	–	–	–
Arginine^*a*^ (Arg)^2^	–	–	14.41	0.009	2.41	0.037	–	–	–	–
Aspartic acid (Asp)^2^	–	–	22.9	0.003	3.62	0.004	–	–	–	–
Cysteine (Cys)^4^	–	–	–	–	–	–	–	–	–	–
Glutamic acid (Glu)^3^	10.33	0.02	17.23	0.006	5.78	<0.001	–	–	–	–
Glycine (Gly)^1^	–	–	–	–	–	–	8.99	0.024	–	–
Histidine^*a*^ (His)^2^	–	–	11.66	0.014	4.79	<0.001	–	–	–	–
Isoleucine^*a*^ (Ile)^1^	–	–	–	–	2.77	0.019	–	–	–	–
Leucine^*a*^ (Leu)^1^	9.22	0.023	–	–	–	–	–	–	–	–
Lysine^*a*^ (Lys)^1,2,3,5^	23.91	0.003	–	–	–	–	–	–	10.88	<0.001
Methionine^*a*^ (Met)^4^	–	–	–	–	3.83	0.002	15.49	0.008	–	–
Phenylalanine^*a*^ (Phe)^1^	10.64	0.017	–	–	–	–	–	–	3.74	0.003
Proline (Pro)^1^	7.43	0.034	–	–	2.36	0.04	–	–	–	–
Serine (Ser)^1^	9.25	0.023	–	–	2.31	0.045	–	–	–	–
Threonine^*a*^ (Thr)^1^	–	–	–	–	–	–	–	–	–	–
Tyrosine (Tyr)^5^	–	–	–	–	–	–	–	–	6.99	<0.001
Valine^*a*^ (Val)^1^	–	–	–	–	–	–	–	–	–	–

Treatments that were not significant (–) and interactive effects that are not displayed were absent from the final models. All dependent variables were log transformed to standardize residuals. Superscript numbers indicate groupings used for PERMANOVA based on correlations (>0.5).

^*a*^ Essential amino acids as defined by Morris (1991).

PCA revealed no clear separation of treatments, although greater variation was seen in plants grown in eCO_2_ at 26 °C. The first two principal components (PC 1 and PC 2) accounted for 62% of the variation in the data set (Supplementary Fig. S1 at *JXB* online). PERMANOVA on correlated amino acid groups, consisting of group 1 (alanine, glycine, isoleucine, leucine, lysine, phenylalanine, proline, serine, threonine, and valine), group 2 (arginine, aspartic acid, histidine, and lysine) and four individual amino acids (glutamic acid, methionine, cysteine, and tyrosine), confirmed patterns observed in individual models (see Supplementary Table S2 for full results), whereby group 1 was significantly affected by CO_2_ and group 2 was significantly affected by temperature (Supplementary Fig, S2A and C, respectively). Temperature and CO_2_ interactively affected all ungrouped individuals.

### Impacts of root damage

**Fig. 4.**
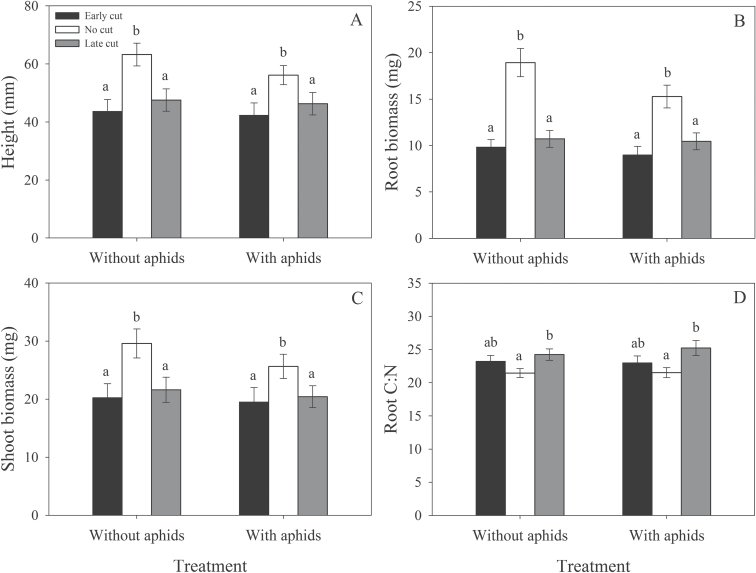
Effects of plant-damage treatment on plant height (A), root biomass (B), shoot biomass (C), and root C:N (D). Mean values (± SE) are shown. Bars with the same letters were not significantly different (*P*<0.05).

Plant-damage treatment had a significant effect on lucerne height (*F*_5,504_=10.73; *P*<0.001), whereby control (uncut) plants were significantly taller than those with damaged roots (Fig. 4A). Similar results were found for both root (*F*_5,496_=22.76; *P*<0.001) and shoot biomass (*F*_5,495_=7.44; *P*<0.001). Overall, early cutting and late cutting reduced root mass by 46% and 37%, respectively (Fig. 4B). Similarly, shoot mass was reduced by 26% and 22% in early and late-cut plants, respectively (Fig. 4C). Significant effects of plant-damage treatment were also seen for root %N (*F*_5,207_=2.58; *P*=0.028), %C (*F*_5,207_=5.05; *P*<0.001), and C:N (*F*_5,207_=3.33; *P*=0.006), whereby control plants had a significantly higher root %N and lower root %C and C:N than late-cut plants. Consistent general increases in C:N were seen across cutting treatments depending on the time at which the plants were cut (Fig. 4D).

**Fig. 5.**
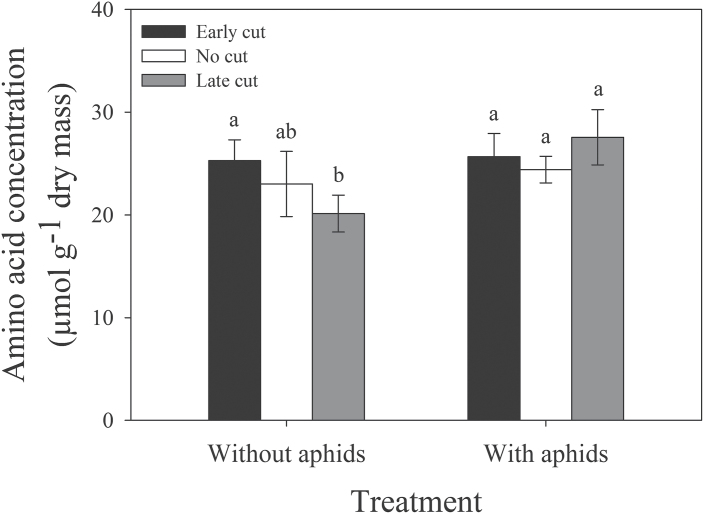
Effects of plant-damage treatment on total foliar amino acid concentrations. Mean values (± SEs) are shown. Bars with the same letters were not significantly different (*P*<0.05).

Plant-damage treatment also significantly affected total amino acids (*F*_1,287_=3.042; *P*=0.011). but had no effect on essential amino acids. On plants without aphids present, total amino acid concentrations were greater in roots that were cut early compared with those cut late, although neither early- or late-cut plants were significantly different from uncut controls, which contained intermediate concentrations. When aphids were present, amino acid concentrations were generally higher, but no significant root-damage effects were detected. However, late-cut plants with aphids contained significantly higher concentrations of amino acids than late-cut plants without aphids (Fig. 5). Similar to individual models (Table 1), glutamic acid and group 2 amino acids were significantly affected by plant-damage treatment (Supplementary Table S2 at *JXB* online). Group 2 amino acids showed dramatic increases in late-cut plants when aphids were present (Supplementary Fig. S2D).

### Interaction effects of environment and root damage

**Fig. 6.**
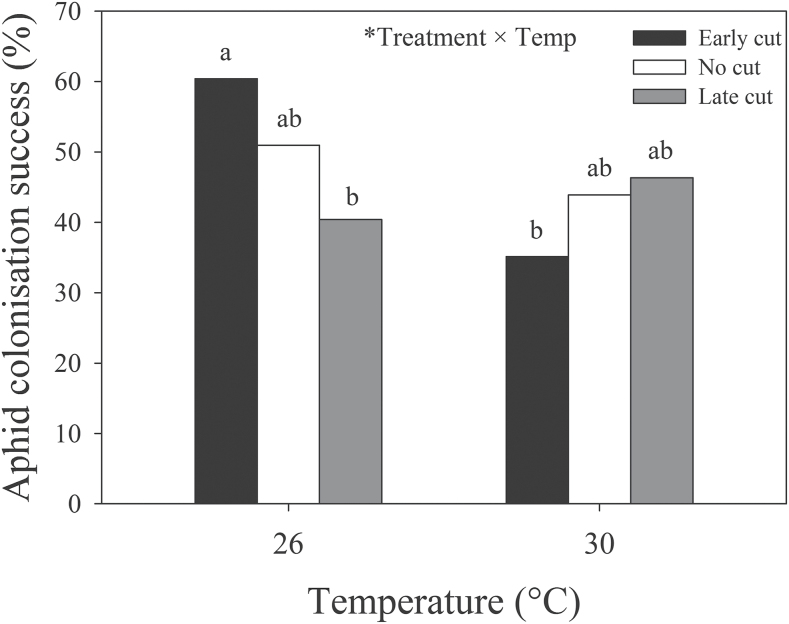
Effects of root cutting on aphid colonization success (i.e. percentage of plants with aphids present upon harvest) under ambient and elevated temperatures. A statistically significant effect is indicated by * (*P*<0.05). Bars with the same letters were not significantly different (*P*<0.05).

**Fig. 7.**
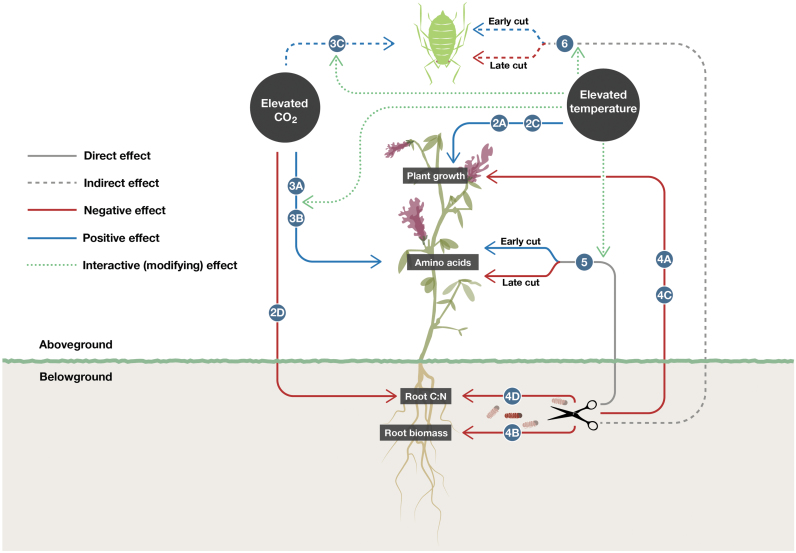
Schematic diagram of the significant positive and negative effects of temperature, CO_2_, and root cutting treatments on plant characteristics and aphids. Numbers and letters correspond to the figures associated with those effects.

Plant-damage treatment and temperature interactively affected lysine, phenylalanine, and tyrosine (Table 1). Specifically, at 26 °C, concentrations of all three amino acids were consistently higher when plants were cut early compared with control plants or plants cut late. Additionally, at 30 °C, concentrations of all three amino acids were significantly higher when late-cut plants were colonized by aphids compared with other treatments. Lysine was strongly correlated with group 1, group 2, glutamic acid, and tyrosine (Supplementary Table S2 at *JXB* online). Plant-damage treatment in interaction with temperature also had a significant effect on aphid colonization success, whereby, at 26 °C, pea aphid colonization success was significantly higher when lucerne roots were cut before aphid inoculation compared with those cut after aphid inoculation, although neither was significantly different from uncut control plants (Fig. 6). Aphids were significantly more successful at colonizing plants with roots that were cut early at 26 °C compared with 30 °C. No significant differences between treatments were seen at 30 °C. Significant effects of different factors and their associated figures are summarized in Fig. 7.

## Discussion

This study is the first to incorporate the effects of both eT and eCO_2_ on the sequence of herbivore arrival within an aboveground–belowground framework. These combined approaches incorporating multiple factors enable greater understanding of how plants and insects will interact in the future (Ryalls *et al.*, 2013*a*). The results demonstrate that eT can counter the positive effects of eCO_2_ and root damage on amino acid concentrations and aphid populations, with important implications for future aphid outbreaks and crop security.

### Impacts of eT and eCO_2_

Expected increases in aboveground biomass and height in response to eCO_2_ were observed at 30 °C, although, at 26 °C, eCO_2_ decreased height and had no effect on biomass. This is probably due to the high numbers of aphids present on the plants under these conditions since data were analysed across all treatments. The decrease in root C:N at eCO_2_ suggests that lucerne increases biological N fixation to compensate for N limitation under these conditions. The hypothesis that elevated CO_2_ stimulates N fixation in legumes is broadly supported (Rogers *et al.*, 2009).

Concentrations of amino acids in group 2 significantly decreased under eT, suggesting that these amino acids were the main temperature response drivers, although no overall decrease in total amino acid concentrations was observed under eT. Total concentrations did, however, increase in response to eCO_2_ at 26 °C, and the amino acids in group 1 were the major drivers of this CO_2_ response. Studies have reported both positive and negative effects of eCO_2_ on amino acid concentrations, although plant type plays an important role in determining the direction of the effect (Docherty *et al.*, 1997; Himanen *et al.*, 2008; Rogers *et al.*, 2009). While tissue dilution effects of eCO_2_ often lead to decreases in amino acid concentrations in plants (Sun and Ge, 2011), legumes often show increases in amino acid concentrations under eCO_2_ due to increases in biological N fixation (Guo *et al.*, 2013). Responses of different cultivars, varieties, or genotypes of the same species to eCO_2_, however, can also vary. For example, Johnson *et al.* (2014) found 86% and 56% increases in essential amino acid concentrations and pea aphid colonization success, respectively, on the cultivar ‘Sequel’ under eCO_2_, whereas essential amino acid concentrations and aphid colonization success decreased by 53% and 33%, respectively, on the cultivar ‘Genesis’ under the same conditions. In other words, some cultivars may become more or less susceptible to aphid attack under climate change conditions, an important consideration for determining future outcomes. Further study combining both eCO_2_ and eT would help to clarify the effects of climate change on aphid responses to different cultivars.

This study used the widely grown public variety ‘Sequel’, with similar results to those found by Johnson *et al.* (2014) using the same cultivar. As with that study, aphid numbers were lower than the reproductive potential of *A. pisum* when feeding on susceptible plants and cultivars (e.g. the lucerne cultivar, ‘Hunter River’). This most probably reflects aphid populations in the field since only cultivars with some aphid resistance are now grown; ‘Hunter River’ was phased out >30 years ago (Ryalls *et al.*, 2013*a*). The exact mechanisms underpinning lucerne resistance to aphids are poorly characterized (Ryalls *et al.*, 2013*a*), but probably include plant traits conferring both antibiosis (e.g. low fecundity) and antixenosis (e.g. inability to colonize) resistance. Evidence was seen for both in the present study. In addition to primary chemistry, secondary chemistry may play a role in lucerne resistance to aphids. In particular, saponins have also been identified as potentially important factors associated with aphid resistance (Goławska *et al.*, 2014). Moreover, the extent to which endosymbionts (obligate and facultative) might help aphids cope with such induced changes in primary and secondary chemistry are unknown in this system, but could also play a role. Simultaneously quantifying both saponins and amino acids, while considering the role of endosymbionts, might therefore provide an important insight into lucerne susceptibility to aphids.

Effects of eT and eCO_2_ on aphids mirrored their effects on amino acid concentrations, suggesting that aphids rely on amino acid concentrations to reproduce and colonize effectively on lucerne. In general, when aphids were present, amino acid concentrations were higher, suggesting that pea aphids promote amino acid metabolism in lucerne to favour population growth. Similar results were found for pea aphid populations fed on N-fixing-deficient *M. truncatula* plants, which had decreased activities of N assimilation-related enzymes and amino acid concentrations under eCO_2_, compared with increases seen in control plants (Guo *et al.*, 2013). Pea aphids performed better on plants grown under eCO_2_ conditions at 26 °C but not at 30 °C. Such negation of eCO_2_ effects by eT has also been shown in other systems (e.g. Murray *et al.*, 2013), demonstrating the importance of considering multiple climate change factors. Both plant amino acid and aphid responses to eCO_2_ were either reduced or negated at 30 °C, suggesting that future temperature increases may have beneficial effects on lucerne by reducing aphid numbers, although this will also depend on how aphid natural enemies are affected by such changes (Ryalls *et al.*, 2013*a*).

### Impacts of root damage and interactions with climate

Aboveground biomass and height of control plants were expectedly higher than those with severed roots. Overall and relative losses in root and shoot mass due to root cutting were similar to the overall reductions in root biomass (36.3%) and aboveground growth (16.3%) that were averaged over 85 experimental studies by Zvereva and Kozlov (2012). Root C:N was higher in plants with severed roots, which is likely to be due to the removal of nodules containing N-fixing rhizobial bacteria and the subsequent impairment of biological N fixation. Root cutting may also have caused carbohydrates to be diverted away from the foliage towards the roots (Blossey and Hunt-Joshi, 2003; Johnson *et al.*, 2011). Pea aphids responded positively to early-cut plants, associated with an increase in amino acid concentrations, but this effect was reversed by eT. One essential amino acid that may be important to consider is lysine, which was strongly correlated with other amino acid groups and closely matched amino acid and aphid responses to different treatments. For example, concentrations of lysine were higher at eCO_2_ (also shown by Johnson *et al.*, 2014) and in early-cut plants maintained at 26 °C. In these conditions, aphids may respond to specific amino acids, including lysine, and even influence their composition in the phloem (e.g. Guo *et al.*, 2013), as suggested by the increase in total amino acid concentration in late-cut plants with aphids present compared with those without aphids present. If aphids are already present on the plant when roots are severed, stress-related increases in foliar N and amino acid concentrations are less likely (Huberty and Denno, 2004) and may be the reason why aphid colonization decreased on late-cut plants at 26 °C. Realistically, lucerne is likely to go through periods of discontinuous stress resulting from variable root damage that would allow aphids to take advantage of periodic increases in foliar N or amino acid concentrations (Huberty and Denno, 2004; Johnson *et al.*, 2012). In this case, changes in temporal patterns of herbivore arrival under future conditions would be important to consider.

Understanding how multiple climate change factors shape crop susceptibility to insect pests is clearly a priority for achieving food security (Gregory *et al.*, 2009), with the amount damaged by insects sufficient to feed 1 billion people (Birch *et al.*, 2011). The present study demonstrates how some aspects of climate change can promote aphid pests, with simulated root herbivory having similar positive effects on the same pests. This could clearly be problematic for plant production. Both these effects, mediated by increases in foliar amino acids, were, however, negated by predicted increases in temperature. Research into future patterns of crop susceptibility to insect pests is at a crossroads (Gregory *et al.*, 2009). Logistic constraints often limit the ability to test multiple climatic factors and multitrophic factors, but, nonetheless, a realistic insight into plant–insect interactions will only be obtained if this challenge is met.

## Supplementary data

Supplementary data are available at *JXB* online.

Figure S1. Principal component analysis of foliar amino acid data with attribute loadings on the first two components PC 1 and PC 2.

Figure S2. Temperature, CO_2_, and plant-damage treatment effects on average amino acid concentrations (mean ±SE) of group 1 and group 2 amino acids.

Table S1. Single-factor treatment effects of CO_2_, temperature, and herbivore damage on individual amino acids (μmol g^–1^ dry mass).

Table S2. Results from multivariate permutational analysis (PERMANOVA) of temperature, CO_2_, and plant-damage treatment effects on different groups of amino acids.

Supplementary Data
