# Supplementary material for: Amino acid-mediated impacts of elevated carbon dioxide and simulated root herbivory on aphids are neutralized by increased air temperatures
Source: J Exp Bot. 2014 Nov 16;66(2):613–23. doi: 10.1093/jxb/eru439 (PMC4286407; doi:10.1093/jxb/eru439)
Supplement: Supplementary Data [file supp_66_2_613__index.html]

Amino acid-mediated impacts of elevated carbon dioxide and simulated root herbivory on aphids are neutralized by increased air temperatures — Amino acid-mediated impacts of elevated carbon dioxide and simulated root herbivory on aphids are neutralized by increased air temperatures — Supplementary Data 

# Amino acid-mediated impacts of elevated carbon dioxide and simulated root herbivory on aphids are neutralized by increased air temperatures

## Supplementary Data

Data files

**Files in this Data Supplement:**

- Supplementary Data - Supplementary Data
