# Supplementary material for: Amino acid-mediated impacts of elevated carbon dioxide and simulated root herbivory on aphids are neutralized by increased air temperatures
Source: J Exp Bot. 2014 Nov 16;66(2):613–23. doi: 10.1093/jxb/eru439 (PMC4286407; doi:10.1093/jxb/eru439)
Supplement: Supplementary Data [file supp_eru439_jexbot131607_file001.pdf]

# Amino acid-mediated impacts of elevated carbon dioxide and simulated root herbivory on aphids are neutralised by increased air temperatures

James M. W. Ryalls[*], Ben D. Moore, Markus Riegler, Andrew N. Gherlenda and Scott N. Johnson

## Supplementary material

**Table S1**: Single-factor treatment effects of $CO_2$, temperature and herbivore damage on individual amino acids (µmol $g^{-1}$ dry mass). Mean values (± standard errors) shown. EC = early-cut no aphids, C = control (no cut) no aphids, LC = late-cut no aphids, ECA = early-cut and aphids, A = no cut and aphids, LCA = late-cut and aphids.

| | $CO_2$ | | Temp (°C) | | Treatment | | | | | |
|---|---|---|---|---|---|---|---|---|---|---|
| Amino acid | a | e | 26 | 30 | EC | C | LC | ECA | A | LCA |
| Alanine (Ala) | 2.276 ± 0.078 | 3.077 ± 0.137 | 2.548 ± 0.110 | 2.774 ± 0.115 | 2.730 ± 0.194 | 2.830 ± 0.238 | 2.220 ± 0.138 | 2.808 ± 0.237 | 3.022 ± 0.179 | 2.277 ± 0.141 |
| Arginine (Arg) | 1.140 ± 0.153 | 2.012 ± 0.390 | 2.182 ± 0.360 | 0.816 ± 0.088 | 2.175 ± 0.450 | 1.602 ± 0.804 | 1.252 ± 0.315 | 1.424 ± 0.427 | 1.019 ± 0.170 | 1.817 ± 0.560 |
| Aspartic acid (Asp) | 4.633 ± 0.244 | 6.433 ± 0.667 | 6.992 ± 0.587 | 3.720 ± 0.206 | 5.239 ± 0.488 | 5.969 ± 1.326 | 3.772 ± 0.393 | 5.757 ± 0.528 | 6.051 ± 0.541 | 5.982 ± 1.212 |
| Cysteine (Cys) | 0.813 ± 0.031 | 0.809 ± 0.030 | 0.817 ± 0.030 | 0.805 ± 0.030 | 0.828 ± 0.041 | 0.768 ± 0.041 | 0.771 ± 0.051 | 0.875 ± 0.048 | 0.846 ± 0.070 | 0.762 ± 0.055 |
| Glutamic acid (Glu) | 1.262 ± 0.039 | 1.493 ± 0.058 | 1.485 ± 0.053 | 1.238 ± 0.041 | 1.390 ± 0.096 | 1.252 ± 0.077 | 1.116 ± 0.063 | 1.355 ± 0.081 | 1.619 ± 0.093 | 1.467 ± 0.081 |
| Glycine (Gly) | 0.372 ± 0.014 | 0.451 ± 0.022 | 0.441 ± 0.019 | 0.372 ± 0.016 | 0.422 ± 0.033 | 0.400 ± 0.031 | 0.365 ± 0.039 | 0.423 ± 0.034 | 0.444 ± 0.025 | 0.398 ± 0.027 |
| Histidine (His) | 0.194 ± 0.017 | 0.316 ± 0.034 | 0.337 ± 0.031 | 0.152 ± 0.013 | 0.241 ± 0.044 | 0.159 ± 0.024 | 0.271 ± 0.041 | 0.281 ± 0.059 | 0.174 ± 0.032 | 0.383 ± 0.059 |
| Isoleucine (Iso) | 0.963 ± 0.036 | 1.117 ± 0.073 | 1.033 ± 0.060 | 1.037 ± 0.049 | 1.006 ± 0.089 | 0.929 ± 0.072 | 0.937 ± 0.095 | 1.048 ± 0.130 | 0.988 ± 0.061 | 1.297 ± 0.112 |
| Leucine (Leu) | 1.326 ± 0.065 | 1.645 ± 0.091 | 1.528 ± 0.083 | 1.415 ± 0.072 | 1.554 ± 0.147 | 1.507 ± 0.140 | 1.351 ± 0.121 | 1.461 ± 0.149 | 1.498 ± 0.121 | 1.477 ± 0.140 |
| Lysine (Lys) | 0.986 ± 0.083 | 1.523 ± 0.136 | 1.274 ± 0.111 | 1.197 ± 0.112 | 1.203 ± 0.148 | 1.147 ± 0.244 | 1.102 ± 0.159 | 0.969 ± 0.103 | 1.094 ± 0.153 | 1.901 ± 0.271 |
| Methionine (Met) | 0.350 ± 0.027 | 0.395 ± 0.026 | 0.410 ± 0.024 | 0.325 ± 0.030 | 0.423 ± 0.053 | 0.238 ± 0.027 | 0.354 ± 0.040 | 0.350 ± 0.044 | 0.416 ± 0.063 | 0.439 ± 0.038 |
| Phenylalanine (Phe) | 0.695 ± 0.047 | 0.977 ± 0.076 | 0.822 ± 0.059 | 0.834 ± 0.067 | 0.809 ± 0.098 | 0.753 ± 0.068 | 0.784 ± 0.100 | 0.787 ± 0.122 | 0.717 ± 0.071 | 1.111 ± 0.160 |
| Proline (Pro) | 2.001 ± 0.229 | 3.341 ± 0.405 | 2.742 ± 0.316 | 2.501 ± 0.332 | 2.773 ± 0.644 | 1.581 ± 0.215 | 2.012 ± 0.409 | 3.490 ± 0.771 | 2.397 ± 0.364 | 3.493 ± 0.696 |
| Serine (Ser) | 1.466 ± 0.057 | 2.040 ± 0.143 | 1.718 ± 0.119 | 1.755 ± 0.087 | 1.889 ± 0.249 | 1.498 ± 0.129 | 1.528 ± 0.185 | 1.963 ± 0.225 | 1.561 ± 0.103 | 1.964 ± 0.169 |
| Threonine (Thr) | 0.745 ± 0.032 | 0.926 ± 0.054 | 0.840 ± 0.043 | 0.818 ± 0.044 | 0.864 ± 0.080 | 0.767 ± 0.067 | 0.714 ± 0.071 | 0.903 ± 0.092 | 0.824 ± 0.053 | 0.900 ± 0.084 |
| Tyrosine (Tyr) | 0.578 ± 0.033 | 0.632 ± 0.042 | 0.547 ± 0.032 | 0.668 ± 0.043 | 0.543 ± 0.050 | 0.568 ± 0.053 | 0.615 ± 0.072 | 0.540 ± 0.061 | 0.600 ± 0.055 | 0.753 ± 0.087 |
| Valine (Val) | 0.998 ± 0.045 | 1.364 ± 0.100 | 1.225 ± 0.085 | 1.106 ± 0.061 | 1.194 ± 0.139 | 1.052 ± 0.106 | 1.023 ± 0.134 | 1.233 ± 0.164 | 1.148 ± 0.078 | 1.360 ± 0.150 |

**Table S2**: Results from multivariate permutational analysis (PERMANOVA) of temperature, $CO_2$ and plant-damage (p-d) treatment effects on different groups of amino acids. For the purpose of PERMANOVA, ungrouped individuals (Glu, Met, Cys and Tyr) were grouped with the highest correlated individual amino acid. * indicates significance (P<0.01) adjusted for absence of mixed effects.

| | df | SS | MS | Pseudo-F | P(perm) |
|---|---|---|---|---|---|
| **Group 1 (Ala, Gly, Iso, Leu, Lys, Phe, Pro, Ser, Thr, Val)** | | | | | |
| Temp | 1 | 0.171 | 0.171 | 2.793 | 0.055 |
| $CO_2$ | 1 | 0.801 | 0.801 | 13.050 | 0.001* |
| P-d treatment | 5 | 0.499 | 0.099 | 1.627 | 0.072 |
| Temp × $CO_2$ | 1 | 0.301 | 0.301 | 4.911 | 0.011 |
| Temp × P-d treatment | 5 | 0.309 | 0.062 | 1.006 | 0.414 |
| $CO_2$ × P-d treatment | 5 | 0.184 | 0.037 | 0.601 | 0.867 |
| Temp × $CO_2$ × P-d treatment | 4 | 0.096 | 0.024 | 0.390 | 0.969 |
| Residuals | 255 | 15.647 | 0.061 | | |
| Total | 277 | 18.008 | | | |
| **Group 2 (Arg, Asp, His, Lys)** | | | | | |
| Temp | 1 | 2.657 | 2.656 | 31.501 | 0.001* |
| $CO_2$ | 1 | 0.290 | 0.290 | 3.442 | 0.025 |
| P-d treatment | 5 | 1.429 | 0.286 | 3.390 | 0.001* |
| Temp × $CO_2$ | 1 | 0.256 | 0.256 | 3.039 | 0.030 |
| Temp × P-d treatment | 5 | 0.580 | 0.116 | 1.374 | 0.145 |
| $CO_2$ × P-d treatment | 5 | 0.401 | 0.080 | 0.950 | 0.450 |
| Temp × $CO_2$ × P-d treatment | 4 | 0.167 | 0.042 | 0.495 | 0.908 |
| Residuals | 255 | 21.504 | 0.084 | | |
| Total | 277 | 27.283 | | | |
| **Group 3 (Glu, Lys)** | | | | | |
| Temp | 1 | 0.721 | 0.720 | 17.234 | 0.001* |
| $CO_2$ | 1 | 0.435 | 0.435 | 10.404 | 0.001* |
| P-d treatment | 5 | 0.612 | 0.122 | 2.930 | 0.004* |
| Temp × $CO_2$ | 1 | 0.258 | 0.258 | 6.167 | 0.004* |
| Temp × P-d treatment | 5 | 0.171 | 0.034 | 0.816 | 0.598 |
| $CO_2$ × P-d treatment | 5 | 0.315 | 0.063 | 1.505 | 0.130 |
| Temp × $CO_2$ × P-d treatment | 4 | 0.048 | 0.012 | 0.288 | 0.975 |
| Residuals | 255 | 10.661 | 0.042 | | |
| Total | 277 | 13.220 | | | |
| **Group 4 (Met, Cys)** | | | | | |
| Temp | 1 | 0.639 | 0.639 | 12.143 | 0.001* |
| $CO_2$ | 1 | 0.103 | 0.103 | 1.953 | 0.124 |
| P-d treatment | 5 | 0.283 | 0.057 | 1.077 | 0.403 |
| Temp × $CO_2$ | 1 | 0.538 | 0.538 | 10.226 | 0.001* |
| Temp × P-d treatment | 5 | 0.161 | 0.032 | 0.612 | 0.810 |
| $CO_2$ × P-d treatment | 5 | 0.233 | 0.047 | 0.885 | 0.559 |
| Temp × $CO_2$ × P-d treatment | 4 | 0.187 | 0.047 | 0.889 | 0.554 |
| Residuals | 255 | 13.412 | 0.053 | | |
| Total | 277 | 15.555 | | | |
| **Group 5 (Tyr, Lys)** | | | | | |
| Temp | 1 | 0.635 | 0.635 | 9.264 | 0.001* |
| $CO_2$ | 1 | 0.497 | 0.497 | 7.248 | 0.002* |
| P-d treatment | 5 | 0.433 | 0.087 | 1.264 | 0.229 |
| Temp × $CO_2$ | 1 | 0.388 | 0.388 | 5.666 | 0.006* |
| Temp × P-d treatment | 5 | 0.426 | 0.085 | 1.242 | 0.216 |
| $CO_2$ × P-d treatment | 5 | 0.201 | 0.040 | 0.588 | 0.878 |
| Temp × $CO_2$ × P-d treatment | 4 | 0.067 | 0.017 | 0.246 | 0.997 |
| Residuals | 255 | 17.471 | 0.069 | | |
| Total | 277 | 20.118 | | | |

**Supplementary material**

**Figure legends**

**Figure S1**: Principal component analysis of foliar amino acid data with attribute loadings on the first two components PC 1 and PC 2. Amino acid abbreviations are denoted in Table 1. Sample scores on PC 1 and PC 2 explain, respectively, 50% and 12% of the variation in the dataset. Plots were coloured according to chamber (i.e. temperature and $CO_2$) conditions (A) and plant-damage treatment (B).

**Figure S2**: Temperature, $CO_2$ and plant-damage treatment effects on average amino acid concentrations (mean ± standard error) of group 1 (A and B) and group 2 (C and D) amino acids. Significant treatment factors according to PERMANOVA indicated * ($P < 0.01$).

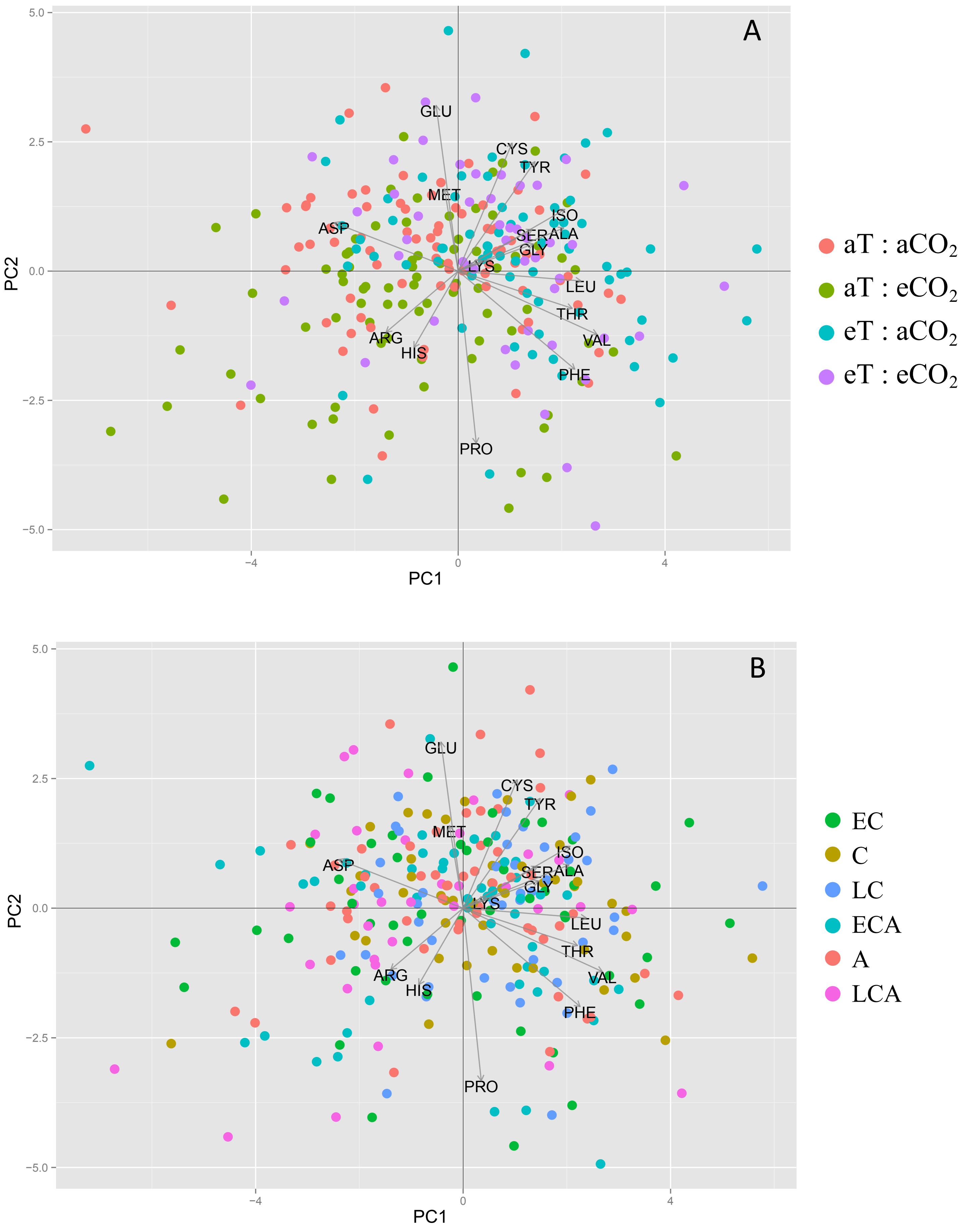
A
PC2
PC1
GLU
CYS
TYR
MET
ISO
ASP
SERALA
GLY
LYS
LEU
THR
ARG
HIS
VAL
PHE
PRO
aT : $aCO_2$
aT : $eCO_2$
eT : $aCO_2$
eT : $eCO_2$
B
EC
C
LC
ECA
A
LCA

Figure S1

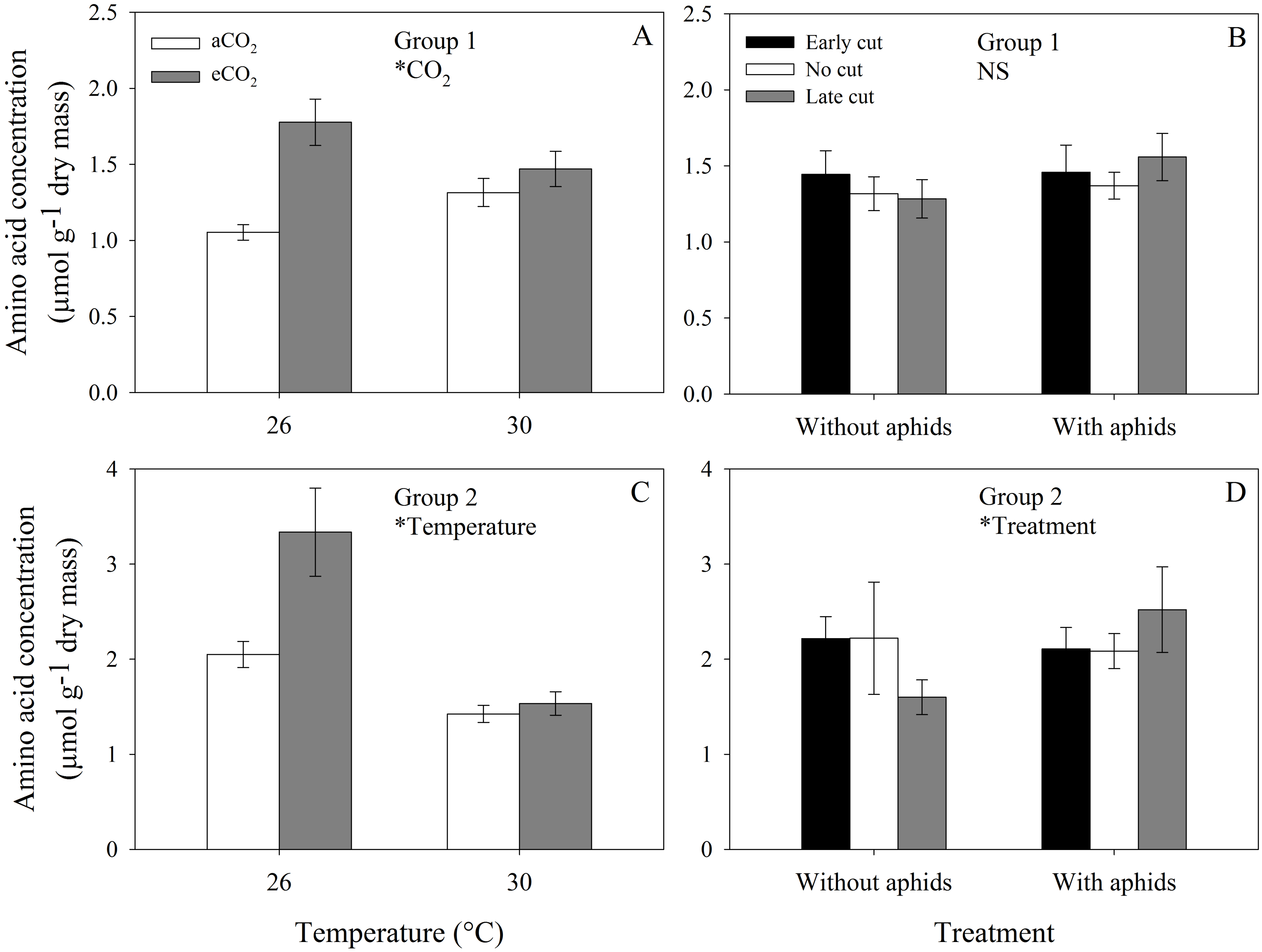
Amino acid concentration (µmol g-1 dry mass)
A
Group 1
*CO2
aCO2
eCO2
B
Group 1
NS
Early cut
No cut
Late cut
C
Group 2
*Temperature
D
Group 2
*Treatment
26
30
Without aphids
With aphids
Temperature (°C)
Treatment

Figure S2
